# Characterizing the Thermal Demands and Mobility Performance During International Wheelchair Rugby Competition

**DOI:** 10.3389/fresc.2022.856904

**Published:** 2022-04-29

**Authors:** Erica H. Gavel, Melissa A. Lacroix, Vicky L. Goosey-Tolfrey, Heather M. Logan-Sprenger

**Affiliations:** ^1^Faculty of Science, Ontario Tech University, Oshawa, ON, Canada; ^2^Canadian Sport Institute Ontario, Toronto, ON, Canada; ^3^School of Sport, Exercise and Health Sciences, Peter Harrison Centre for Disability Sport, Loughborough University, Loughborough, United Kingdom; ^4^Faculty of Health Science, Ontario Tech University, Oshawa, ON, Canada

**Keywords:** wheelchair rugby, thermoregulation, wheelchair mobility, Paralympic sport, performance

## Abstract

**Objective:**

To determine the thermoregulatory responses and mobility performance of wheelchair rugby (WCR) players during international competition.

**Methods:**

Eleven male National Team WCR players volunteered for the study. Testing occurred during a four game series against international competition (temp 24.7 ± 0.7°C, relative humidity 50.1 ± 3.6%), with movement time (MT) and gastrointestinal temperature (T_gi_) recorded continuously.

**Results:**

The mean maximal T_gi_ was 38.6 ± 0.6°C (37.9–39.7) and did not significantly differ among Low-Class, Mid-Class, and High-Class athletes (*p* > 0.05). Moreover, there was a strong and significant relationship between minutes (min) played per quarter of the game and change in T_gi_ (*r* = 0.36, *p* = 0.01). Athletes moved a total of 27:43 ± 9:40 min:seconds (s), spent a total of 15:02 ± 8.23 min:s in Zone 1 (53.5%), 8:19 ± 3:20 min:s in Zone 2 (31.7%), and 5:59 ± 1:51 min:s in Zone 3 (21.3%). There were no differences among classification in total movement time (*p* = 0.169) or for speed in Zone 1, Zone 2, or Zone 3 (*p* > 0.05). The relationship between peak forward speed and total movement time was strong (*p* = 0.021, *r* = 0.68).

**Conclusion:**

This study demonstrated that the time spent in absolute movement zones is not classification dependent, the change in core temperature is related to movement time per quarter. Furthermore, peak speeds obtained on-court were linked to overall movement time which suggests athletes should warm-up before going on court.

## Introduction

Wheelchair rugby (WCR) is an intermittent contact sport (1) which spends most time at low speeds (<50% of mean peak speed) (2, 3). In WCR, there are a total of seven classifications ranging from, 0.5 to 3.5 in order of greatest impairment to least impaired (i.e., 0.5, cervical spinal cord injury (cSCI); 3.5, quad-amputee). During an official World Wheelchair Rugby (4) game which consists of four 8-min quarters with stopped time, the total number of points on court permitted at any time is 8.0 (*n* = 4 players) (4). With that, the demands of the game and activity profiles can be dependent upon classification (5, 6), e.g., Rhodes et al. (2), clearly showed that WWR Class 0.5 athletes significantly traveled less and had lower peak speeds relative to Class 3–3.5, 881 ± 137 m vs. 1153 ± 172 m and 3.0 vs. 3.8 m/s, respectively.

While WCR is competed indoors in a temperature-controlled gymnasium (18–20°C), it has been demonstrated in athletes with a cSCI (7, 8) and without a SCI, that exercising in thermoneutral environments may induce severe heat strain (9–11). For example, work by Griggs et al. (9) reported that WCR athletes with a cSCI reached a core temperature of 39.3 ± 0.5°C, whereas athletes without a SCI reached 38.8 ± 0.3°C. Moreover, in other wheelchair sports Logan-Sprenger and McNaughton (10), noted two athletes from the Canadian Senior Women's National wheelchair basketball team without a SCI were attaining (>39.3°C) which were sustained for 10–25 mins over multiple games. As such, while level of injury can influence ones' ability to regulate body temperature, people should also be mindful that athletes without SCI's may also experience heat strain.

During exercise, metabolic energy is converted to one of two forms, mechanical energy to perform external work (20–30%), or thermal energy to produce heat (70–80%) (12). That said, if heat production exceeds heat dissipation, one will experience increases in core body temperature and decrements in physiological performance (13, 14). For example, Forsyth et al. (15) found that when the metabolic heat production was similar among athletes with cSCI, paraplegia, and non-SCI, those with cSCI displayed the greatest compromised sweat response. Moreover, similar outcomes were observed in work by Griggs et al. (9) where the level of injury was correlated with changes in core temperature and heat storage.

Although sport practitioners and coaches should be mindful that the level of SCI can be correlated to heat storage and increases in core temperature, one should also consider the influence overall playing time has on heat storage and potential detriments in performance. For example, although the work by Griggs et al. (16) demonstrated that athletes with a cSCI reported higher core temperatures than non-SCI athletes, this work was conducted in a practice setting where the athletes played the entire simulated game. Extending this work but within wheelchair basketball, Logan-Sprenger and McNaughton (10) gathered data during international competitive game play to which, the will-to-win was high, providing high ecological validity. As such, the intensity of play along with coach substitutions influencing playing time may be an important difference between a simulated game and a vital game for international ranking. As of late, no study has characterized mobility performance and examined the influence these demands may have on core body temperature, thermoperception, and peak speed during international WCR competition. Thus, the purpose of this study was to (1) characterize mobility performance, (2) describe the thermoregulatory responses, and (3) evaluate the physiological and thermoperception of international level WCR players during a World Wheelchair Rugby event against an international Top 10 ranking competitor.

## Methods

### Subjects

Eleven (*n* = 11; cSCI = 10, quad amputee = 1) elite WCR players volunteered to participate in the study (see Table 1). The research team tested 3–4 athletes per game with each athlete being monitored once. As such, data collection occurred over four separate games to ensure all 11 athletes were tested. All participants were male and members of the Canadian National WCR Team. Typically, the team had three athletes with a cSCI, and one quad amputee on the floor at the same time. Each player had played at the international level for 10.5 ± 6.5 yrs and trained an average of 10–15 h per week. Participants were informed of the experimental protocol before written informed consent was obtained. All procedures were approved by the Ontario Tech University Ethics Committee (file #16414) and conformed to the principles defined in the Declaration of Helsinki.

**Table 1:** Descriptive characteristics of the male participants (mean ± SD).

	**IWRF class**	**Level of injury (LOI)**	**Completeness of lesion**	**Pre-USG**	**Total PT (mins:secs)**	**Δ Tc (**°**C)**	**Mean Tc (**°**C)**	**Peak Tc (**°**C)**	**Mean HR (bpm)**	**Peak HR (bpm)**
**SCI**										
	0.5 1.0	C6 C5	Complete Complete	1.001 1.008	17:38 19:27	0.6 1.4	38.5 38.7	38.9 39.0	83 106	91 124
	1.0	C6	Incomplete	1.010	9:30	1.4	37.5	37.9	89	115
	1.5	C7	Complete	1.010	5:46	1.4	37.9	38.3	96	130
	2.0	C6	Incomplete	1.006	7:42	1.1	37.7	38.1	86	119
	2.0	C6	Complete	1.013	10:42	1.4	38.2	38.6	95	110
	2.0	C6	Incomplete	1.005	11:50	2.3	38.8	39.7	110	135
	2.0	C6	Incomplete	1.006	12:21	0.9	38.0	38.2	94	104
	3.0	C6	Incomplete	1.017	18:09	0.6	38.0	38.3	120	156
	3.0	C7	Complete	1.014	10:59	0.9	37.7	38.1	98	116
	3.5	QA	NA	1.013	23:25	0.7	38.5	38.8	134	176
										
***Mean***				1.010	12:38	1.2	38.2	38.6	99	123
***SD***				0.003	6:40	0.5	0.5	0.7	10	16

*LOI, level of injury; QA, quad-amputee; BM, body mass; USG, urine specific gravity; PT, playing time; **Δ** Tc, change in core temperature from start to end of game; Mean Tc, core temperature from start to end of game; HR, heart rate*.

### Design

This was an observational study assessing wheelchair mobility performance, gastrointestinal temperature (T_gi_), heart rate (HR), ratings of perceived exertion (RPE, Borg 6–20), thermal sensation (TS), and thermal comfort (TC) during a four game series against international competition at the 2019 Japan World Wheelchair Rugby Challenge.

### Methodology

Upon waking on game day athletes were asked to provide a mid-stream urine sample for measurement of urine specific gravity (USG) (17) using a handheld refractometer (Atago–PEN-PRO, Geneq, Montreal QC) (17). Additionally, athletes were instructed to swallow an ingestible thermistor (e-Celcius; Bodycap; Herouville Saint-Clair, France) a minimum of 6 h before the game to measure gastrointestinal temperature (18, 19).

Athletes arrived at the gymnasium at their usual pregame time (~2 h prior to the game); 1 game took place at 12:00 (noon) while the other 3 games started at 18:00. The athlete's water bottle(s) was labeled with the athletes' name and weighed prior to and upon completion of the game to determine total fluid intake throughout the game. Three athletes had a slushie drink following warm-up and before the start of the game and used a water spray during the match (Low-Class, *n* = 1; Mid-Class, *n* =2 ). T_gi_ was continuously measured and recorded throughout the game from the start of warm-up until the end of the game and downloaded intermittently. HR was collected throughout using a downloadable Polar® OH1 heart sensor (Polar, CAN). Each athlete's chair was equipped with an inertial measurement unit (IMU) (Shimmer, Cambridge MA) positioned on the axels and center of chair frame (20) to measure peak speed, real-time speed, and time spent in different speed zones which was recorded throughout the game. Speed zones were categorized as: Zone 1 = 0–1 m/s, Zone 2 = 1–2 m/s, Zone 3 = > 2 m/s. Moreover, playing time and movement time were also recorded. Playing time was characterized as time defined by the “game clock” without the inclusion of stoppages, whereas movement time was the total time the athlete was on-court. For classification and positional analysis, athletes were grouped as Low-Class to (0.5–1.5), Mid-Class (2.0–2.5), and High-Class (3.0–3.5) (4). RPE (21), TS (22), and TC (22) were collected upon substitution or at the completion of each quarter.

### Statistical Analysis

All data was tested for normality of distribution and displayed as the mean and standard deviation. Differences between quarters were analyzed using a one-way ANOVA, and time verses group was tested using a mixed-ANOVA, to detect singular differences, a Tukey's honestly significant difference (HSD) *post-hoc* was performed. A Student's paired *t-test* was used to compare singular parameter differences where appropriate. Categorical data was tested using the Krusal-Wallis test, to detect singular differences a Dunn's *post-hoc* test was performed. A Wilcoxon Signed Ranks Test was detected to test differences in singular categorical data. Statistical significance was accepted at *p* < 0.05. Correlations between variables were assessed using a Pearson's correlation analysis. Exact *p*-values, Cohen's D, and 95% confidence intervals are presented to show magnitude of effect. The magnitude of effect was classed as trivial (<0.2), small (0.2-0) moderate (0.6–1.2), large (1.2–2.0), and very large (≥2.0) (23).

## Results

### Ambient Conditions

The gymnasium temperature (°C) and relative humidity (RH) (%) was similar between games and remained stable within games (pre 24.6 ± 0.8°C, RH 51.4 ± 2.4%; post, 24.4 ± 0.1°C, RH 50.4 ± 3.0%, *p*>0.05).

### Morning Urine Specific Gravity and Fluid Intake

All athletes were hydrated on game day (Table 1) with a mean USG of 1.010 ± 0.004 (1.005–1.015). On average, athletes consumed 1073 ± 840 milliliters (ml) (112–1,669 ml) of fluid per game.

### Game Playing Time

There was large variability in playing time among athletes (2:58–23:25 min:s). On average, athletes played a total of 14:04 ± 5:49 min:s (Table 1). There were no differences in playing time among Low-Class, Mid-Class, and High-Class players (*p* = 0.169), and three athletes played >50% (>16 min) of the game (Low-Class: 19:27; High-Class: 18:09 min:s; High-Class: 23:25 min:s).

### Movement Time and Time Spent in Velocity Zones

**Figure 1:**
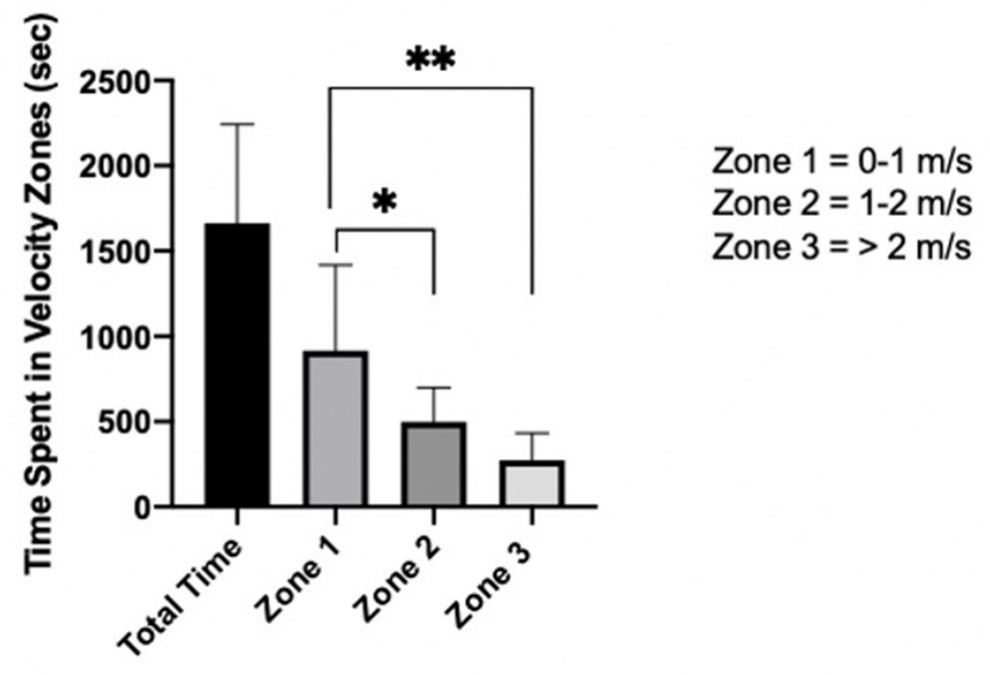
Mean movement time [seconds (sec)] spent in each velocity zone (zone 1–3) throughout a wheelchair rugby game. (*) denotes significance between Zone 1 and Zone 2, whereas (**) denotes significance between Zone 1 and Zone 3.

Excluding warm-up, athletes moved a total of 27:43 ± 9:40 min:s (12:10–43:10) over the course of the game (Table 2). On average, athletes spent a total of 15:02 ± 8.23 min:s in Zone 1 (53.5%), 8:19 ± 3:20 min:s in Zone 2 (31.7%), and 5:59 ± 1:51 min:s in Zone 3 (21.3%) (Figure 1). Movement time among Low-Class, Mid-Class, and High-Class did not significantly differ (*p* = 0.169) with athletes playing a mean of 31:27 ± 9:27, 25:48 ± 10:22, 25:18 ± 11:27 min:s, respectively.

**Table 2:** WC kinematics throughout the WC game.

	**IWRF class**	**Injury**	**Peak speed (m/s)**	**Movement time (mins)**	**Total distance (m)**	**Zone 1 (m)**	**Zone 2 (m)**	**Zone 3 (m)**
	0.5	C6	2.59	26.1	3177.2	652.1 (19.8%)	1713.4 (51.8%)	0 (0%)
	1.0	C5	3.4	43.0	2484.9	423.9 (17.1%)	1139.7 (45.9%)	887.4 (4.0%)
	1.0	C6	3.6	37.4	1249.6	1079.7 (86.4%)	153.4 (12.3%)	16.6 (1.3%)
	1.5	C7	3.5	22.0	1449.0	212.3 (14.7%)	653.4 (45.1%)	548.6 (37.9%)
	2.0	C6	4.5	34.7	1905.8	377.3 (19.8%)	992.4 (52.1%)	534.8 (28.1%)
	2.0	C6	3.1	27.3	1541.2	330.6 (21.5%)	771.8 (50.1%)	407.3 (26.4%)
	2.0	C6	2.8	26.4	1884.5	266.4 (14.4%)	759.5 (40.3%)	774.0 (41.1%)
	2.0	C6	4.1	43.4	1316.2	212.0 (16.1%)	653.0 (49.6%)	411.3 (31.2%)
	3.0	C7	3.4	15.6	1055.6	180.1 (17.1%)	543.8 (51.5%)	320.2 (30.3%)
	3.0	C6	4.0	21.4	795.4	264.4 (18.2%)	715.5 (49.4%)	469.9 (32.4%)
	3.5	QA	5.3	37.5	3436.8	322.9 (9.4%)	1144.0 (33.3%)	1379.6 (40.1%)
								
***Mean***			3.8	27.4	1857.0	384.4 (23.4%)	814.7 (44.3%)	466.3 (25.6%)
***SD***			0.77	9.4	877.1	269.1 (21.2%)	419.9 (12.3%)	383.8 (13.9%)

*Zone 1, 0-1 m/s; Zone 2, 1-2 m/s; Zone 3, > 2 m/s; m, meters; s, seconds; %, percent of total distance spent in each velocity zone*.

**Figure 2:**
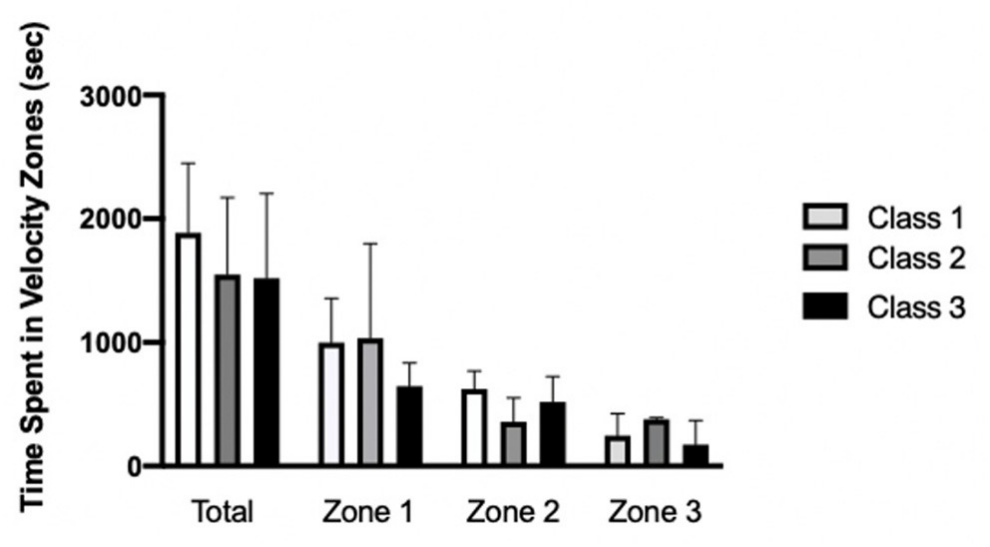
Mean time spent in different velocity zones (Zones 1–3) during a wheelchair rugby game and movement time for Class 1 (*n* = 4), 2 (*n* = 4), and 3 (*n* = 3). *p* > 0.05.

There was no difference among classification in total movement time (*p* = 0.169) or for Zone 1 (*p* = 0.601), Zone 2 (*p* = 0.173), or Zone 3 (*p* = 0.222) (Figure 2). On average, time spent in Zone 1 was significantly greater than Zone 2 (*p* = 0.0154, 95% CI 74.4 to 757.4, ES = 1.09) and Zone 3 (*p* = 0.0002, 95% CI 298.7 to 981.4, ES = 1.53), whereas Zone 2 and Zone 3 did not significantly differ (*p* = 0.253, 95% CI −117.2 to 565.9, ES = 0.87).

There was a significant difference in cumulative distance between Zone 1 and Zone 2 (Zone 1, 384.4 ± 269.1 vs. Zone 2, 814.7 ± 419.9 m, *p* = 0.025, 95% CI −812.2 to −48.3, ES =1.22), while Zone 1 vs. Zone 3 (Zone 1, 384.4 ± 269.1 vs. Zone 3, 466.3 ± 383.8 m, *p* = 0.858, 95% CI −463.8 to 300.1, ES = 0.25) and Zone 2 vs. Zone 3 did not differ (Zone 2, 814.7 ± 419.9 vs. Zone 3, 466.3 ± 383.8 m, *p* = 0.079, 95% CI −33.55 to 730.44, ES = 0.87) (Table 2).

Peak speed in the game did not significantly differ between classification (Low-Class, 3.24 ± 0.45 m/s; Mid-Class, 4.44 ± 1.14 m/s; High-Class, 4.22 ± 0.94 m/s; *p* = 0.373). The mean peak forward speed throughout games was 3.58 ± 0.9 m/s. There were no significant differences in peak speed between quarter 1, quarter 2, quarter 3, and quarter 4 (*p* = 0.338). Furthermore, there were no significant differences in time spent in Zone 1, Zone 2, and Zone 3 between quarters (*p* > 0.05).

### Thermoregulatory Responses Across the Game

**Figure 3:**
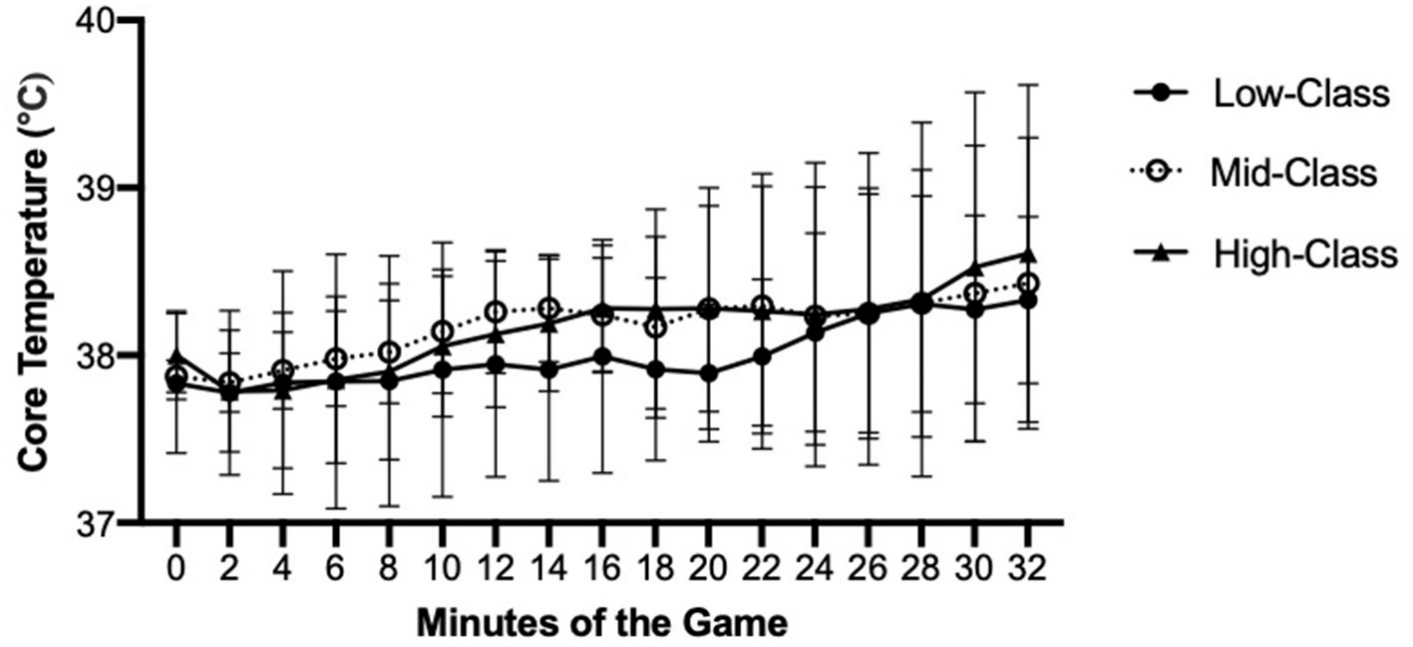
Mean gastrointestinal temperature among Low-Class, Mid-Class, and High-Class athletes across a WCR game.

Mean T_gi_ was significantly greater from the start (following warm-up) to end of game (start, 37.9 ± 0.3°C vs. finish, 38.6 ± 0.7°C, *p* = 0.009). Mean T_gi_ significantly differed between quarter 1, quarter 2, quarter 3, and quarter 4 (*p* < 0.05**)**. The mean rise of T_gi_ was 1.2 ± 0.5°C, and there was no significant rise in T_gi_ between quarter 1, quarter 2, quarter 3, and quarter 4 (*p* = 0.766). The mean maximal T_gi_ was 38.6 ± 0.6°C (37.9–39.7). T_gi_ did not significantly differ among Low-Class, Mid-Class, and High-Class athletes throughout the game (*p* > 0.05, Figure 3). Furthermore, there were no significant differences between athletes with complete and incomplete SCIs (*p* > 0.05). Two of the 11 athletes (Low-Class; Mid-Class reached a T_gi_ of >39°C and at the start of quarter 4 sustained it for 14:02 ± 1:06 min:s (13:15; 14:48) with a mean movement time of 30:10 ± 4:08 min:s (27:15; 33:05) and playing time of 14:00 ± 2:59 min:s. (19:27; 11:50).

### Thermal Sensation, Thermal Comfort, Ratings of Perceived Exertion

The median TS for the game was 6 (1). TS did not significantly differ from the start to end of game [start, 6 (2) vs. end, 5 (2), *p* = 0.930]. The median TS did not significantly differ between quarters (*p* = 0.210) or between classifications (*p* = 0.178). As well, median TC was 2 (1). TC did not significantly differ from start to end of game [start, 2 (2) vs. end, 3 (2), *p* = 0.586]. TC did not significantly differ between quarters (*p* = 0.750) or among classification (*p* = 0.359). The median RPE was 15 (2). RPE significantly increased from the start to end of game [start, 13.0 (8) vs. end, 15.0 (2.5), *p* = 0.023]. The median RPE did not significantly differ between quarters (*p* = 0.875) or between classification (*p* = 0.951).

### Correlations

**Figure 4:**
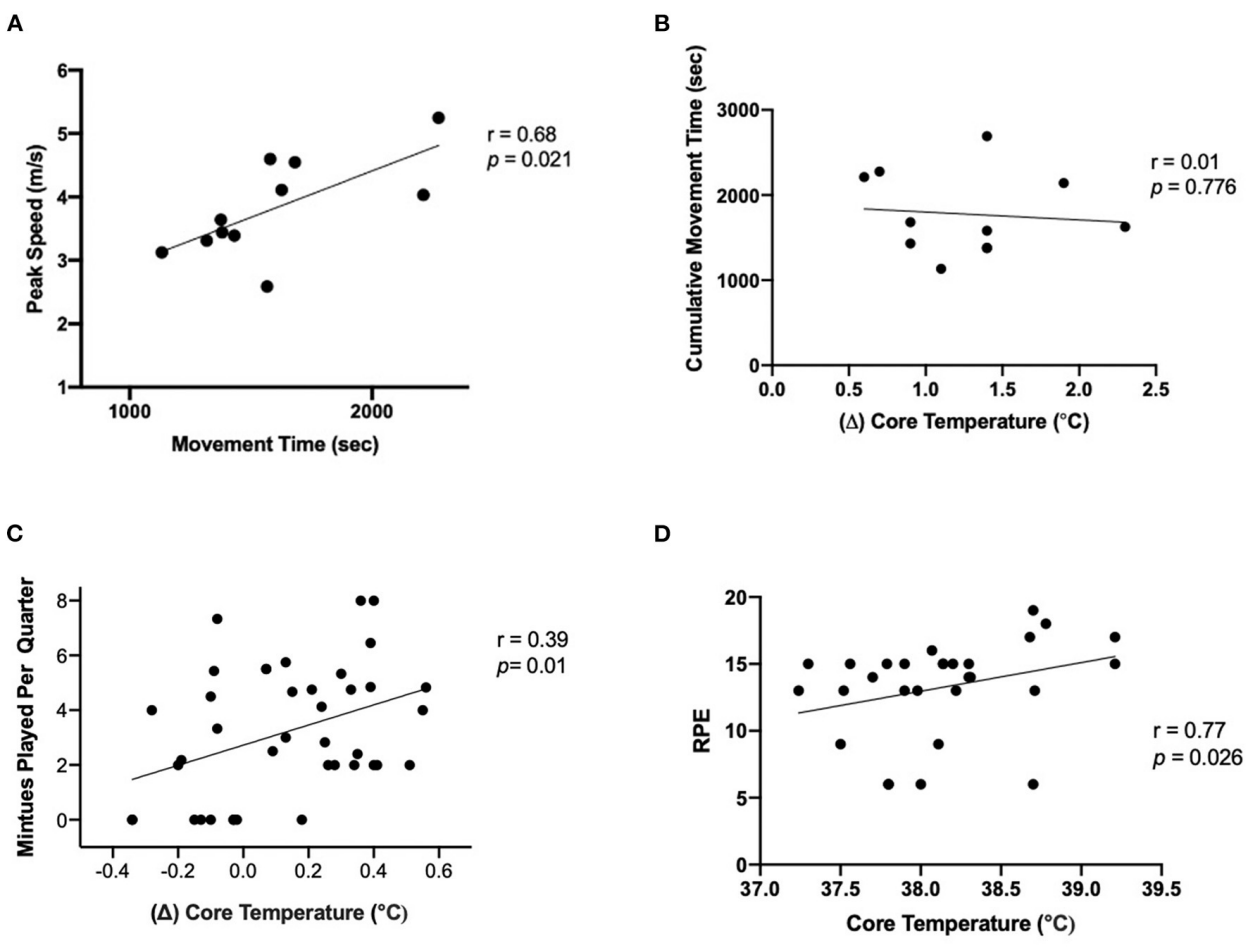
**(A)** correlation between peak speed (m/sec) and movement time, **(B)** correlation of mean (Δ) of gastrointestinal temperature and cumulative movement time (sec), **(C)** correlation of mean change (Δ) of gastrointestinal temperature (°C) and playing time per Q (mins), and **(D)** correlation of gastrointestinal temperature (°C) and RPE (6–20).

There was a significant relationship between total movement time and time spent in Zone 1 (*p* = 0.002, *r* = 0.85); however, the relationship between total movement time and Zone 2 (*p* = 0.315, *r* = 0.35), and Zone 3 (*p* = 0.51, *r* = 0.24) was not strong. Moreover, the relationship between peak forward speed and total movement time per quarter was strong (*p* = 0.021, *r* = 0.68, Figure 4A).

There was not a significant relationship between total playing time and change in T_gi_ (*p* = 0.641, *r* = 0.41), change in T_gi_ and time spent in Zone 1 (*p* = 0.236, *r* = 0.19), Zone 2 (*p* = 0.112, *r* = 0.26), and Zone 3 (*p* = 0.134, *r* = 0.24), and peak T_gi_ and total movement time (*p* = 0.245, *r* = 0.438, Figure 4B). However, there was a strong relationship between movement time and change in T_gi_ per quarter (*p* = 0.014, *r* = 0.39, Figure 4C). There was a strong relationship between T_gi_ and RPE (*p* = 0.026, *r* = 0.77, Figure 4D) while the relationship between T_gi_ and TC (*p* = 0.341, *r* = −0.15) and TS (*p* = 0.61, *r* = −0.08) was not significant.

## Discussion

This is the first study in WCR to demonstrate that differences in core temperature may not be related to classification or physiological function, but with movement time per quarter. Furthermore, the results of this study also exhibited, (1) the mean movement time was 28.3 ± 8.5 min and did not significantly differ among classification, (2) athletes spent most of their time at low speeds (Zone 1= 0–1 m/s) during the game, (3) there was a positive relationship between movement time and peak speed (*r* = 0.68, *p* = 0.021), (4) there were no significant differences among core body temperature and classification, and (5) TS and TC did not change over the course of the game.

### Wheelchair Mobility Profiles

The present study supported previous findings suggesting that athletes spend most time in low intensity zones; 53.5% in Zone 1, 31.7% in Zone 2, and 21.3% in Zone 3 (1, 11, 12). That said, contrary to Rhodes et al. (1), we did not display any significant differences between classification groups, which may be due to a smaller sample size and competitor discrepancies (24) (i.e., Zonal Championships vs. World Wheelchair Rugby Challenge). Across the game, we found no differences in mean peak speed between quarters which was also noted by Rhodes et al. (1). Interestingly, there was a strong correlation between movement time and peak speed, which warrants further investigation suggesting that warming-up courtside prior to being substituted into the game should be encouraged allowing athletes to achieve high wheelchair speeds when they are not the starting players. This is a topic of recent study (24) and needs to be considered alongside individual thermoregulatory responses.

### Thermoregulatory Responses

Notably, the present study demonstrated a positive relationship between playing time per quarter and change in core temperature with no differences in core temperature between classification, which aligns with wheelchair basketball work done by Logan-Sprenger and McNaughton (10). In the study, the researchers demonstrated no core temperature difference between classification, instead they observed that the change in core temperature was dependant on minutes played, which is like the present study showcasing that the change in core temperature was dependent on minutes moved per quarter. Although, it is well established that sudomotor activity plays a pertinent role in thermoregulation (25), both the present study and the one by Logan-Sprenger and McNaughton (10) demonstrate that in team sports such as WC basketball and rugby, the increase in core temperature may be more dependent on playing time rather than muscular function and the inability to dissipate heat; however, more research is needed to better understand the relationship (26).

While the present study saw ~1.2°C change of core temperature over the course of the game, there were no differences among classification and only two athletes who reached >39°C and sustained it for ~14 min. This data, however, contrasts with work published by Griggs et al. (9) who demonstrated significant differences between classification. In the study by Griggs et al. (9), the authors reported that lower class players displayed an increase of 1.6°C and upper-class players showed an increase of 0.7°C. In contrast to our methodology, Griggs et al. (9) collected data during an intra-squad game where the athletes played the entire game under simulated game conditions. Given that the current study was against an international competitor at a World Wheelchair Rugby event, there was high variability in playing time (CV = 36%) as the team was playing to win. As such, it is hard to compare T_gi_ responses between studies given that playing time and competition level differed (27).

### Thermo-Perception

The current study demonstrated no correlation between core temperature and thermal sensation or comfort. Furthermore, there were no differences in thermal sensation or comfort from the start to end of the game, between quarters, or among classification groups, which is in line with work by Webborn et al. (28) and Griggs et al. (25). In the study by Webborn et al. (28), participants completed an intermittent sprint protocol with pre-cooling, per-cooling, and no cooling. In the study, there was no significant relationship between core temperature changes and thermal sensation. Furthermore, similar results were exhibited by Griggs et al. (25) who analyzed the influence relative humidity had on heat storage for athletes with a SCI. In the study, there was no significant relationship between increases in core temperature and changes in thermal perception.

Thermo-physiological models have been developed to assess the influence of environmental conditions on the human body (29, 30); however, no model has been validated with the inclusion of exercise in athletes with a SCI or an impairment. For example, work by Flouris and Cheung (31) has demonstrated that thermoperception can vary for the same stimulus of skin and core body temperature during exercise in a hot environment. Furthermore, research by Nicotra and Ellaway (32) suggests that the level and completeness of the SCI can influence heat and cold thresholds for a similar stimulus, whereas work by Griggs et al. (25) reported no difference between paraplegic and tetraplegic in similar environmental conditions. Moreover, work by Webborn et al. (28) demonstrated that thermal sensation did not correlate to changes in core temperature or total time exercising.

## Limitations

Although all athletes but one had a SCI, it's worth highlighting that we selected to include a player with quadruple amputation in the data analyses. Close inspection of our findings indicates that removal of the amputee would not impact the results too much as the athletes were grouped on a positional basis verses physiological function. Furthermore, their thermoregulatory responses were within 1 SD of the group responses. Secondly, given that the data was collected over a series of four separate games, one could suggest that certain games could be lower intensity than others. However, we observed no difference in RPE from one game to the next.

## Conclusion

To our knowledge, no study has evaluated the relationship between activity profiles, thermoregulatory responses, and thermo-perception during international WCR match play. First off, this study demonstrated that the time spent in absolute movement zones is not classification dependent, the change in core temperature is related to movement time per quarter. Secondly, peak speeds obtained on-court were linked to overall movement time which suggests a mid-event warm-up could be beneficial. Finally, like previous work, thermo-perception models should be taken with caution when working with athletes with a SCI.

## Data Availability Statement

The original contributions presented in the study are included in the article/supplementary materials, further inquiries can be directed to the corresponding author.

## Ethics Statement

The studies involving human participants were reviewed and approved by Ontario Tech University Research Ethics Board. The patients/participants provided their written informed consent to participate in this study.

## Author Contributions

All authors provided substantial contributions to the conception, study design, and the drafting of the work, or revising it critically. Final approval of the version submitted/published and consent for publication has been agreed by all authors.

## Funding

EG was supported in this original investigation by funding from Mitacs and Own the Podium.

## Conflict of Interest

The authors declare that the research was conducted in the absence of any commercial or financial relationships that could be construed as a potential conflict of interest.

## Publisher's Note

All claims expressed in this article are solely those of the authors and do not necessarily represent those of their affiliated organizations, or those of the publisher, the editors and the reviewers. Any product that may be evaluated in this article, or claim that may be made by its manufacturer, is not guaranteed or endorsed by the publisher.
